# What’s that you’re eating? Social comparison and eating behavior

**DOI:** 10.1186/s40337-017-0148-0

**Published:** 2017-04-27

**Authors:** Janet Polivy

**Affiliations:** University of Toronto, Toronto, Canada

**Keywords:** Social comparison, Eating, Social influence, Restrained eaters

## Abstract

People seem to have a basic drive to assess the correctness of their opinions, abilities, and emotions. Without absolute indicators of these qualities, people rely on a comparison of themselves with others. Social comparison theory can be applied to eating behavior. For example, restrained eaters presented with a standard slice of pizza ate more of a subsequent food if they thought that they had gotten a bigger slice of pizza than others (i.e., had broken their diets), whereas unrestrained eaters ate less. Social influences on eating such as modeling and impression formation also rely on comparison of one’s own eating to others. Comparing one’s food to others’ meals generally influences eating, affect, and satisfaction.

## Plain English Summary

People seem to have a basic drive to assess the correctness of their opinions, abilities, and emotions. Without absolute indicators of these qualities, people rely on a comparison of themselves with others. This is called social comparison, and it applies to eating as much as to other behaviors. Research shows that people compare what others are eating to what they themselves eat, and draw conclusions from these comparisons about how much they should eat, whether they have eaten a normal amount or overeaten, how they feel about what they ate, and how satisfied they are with themselves and their food.

## Background

Research shows that when we eat with someone else, we are likely to eat similarly to that person, modeling or mimicking what and how much the other person eats (see e.g., [1] for a review). This effect is well-demonstrated and reasonably well-known, but it turns out that food eaten by other people has many effects on us. For example, research has demonstrated: the trust-inducing effects of eating the same food as a stranger with whom we share a meal [2]; that we eat more [3] and enjoy it more when eating with other people [4]; and that even infants associate people who go together in ways such as wearing the same clothes or interacting in positive ways with eating the same foods [5].

One interesting aspect of this work is the implication it suggests that people eating with others are extremely sensitive to what the others are eating. Obviously, the people in the studies just described, even infants, were noticing and reacting to the foods served not only to themselves, but to their eating companions. This social comparison of how others’ meals compare to one’s own food is what this paper will discuss.

## Main text

The social psychology literature on what is called Social Comparison shows that people compare themselves to others on a wide variety of dimensions. We want to assess the correctness of our opinions, abilities, and emotions, and so we look to how others act in order to ascertain where we fall [6]. Sometimes we make comparisons with people who are in a better position than we are, “upward comparisons” (e.g., [7]), in order to determine how attainable these better positions are, although this often leaves us feeling badly when it seems we cannot achieve these higher levels. We also compare ourselves to those in less advantageous positions, “downward comparisons” (e.g., [8]), which allow us to feel good about ourselves or at least better. It turns out, we do this sort of comparing with others for our food consumption as well as these other factors.

We even use our food consumption, and our knowledge that everyone compares each other on this dimension, to affect how others perceive us. For example, Mori, Chaiken, and Pliner [9] demonstrated that when women want to make a positive impression on others, they eat less than when they are not concerned with impressing someone. Vartanian and his colleagues [10] reviewed a whole literature that examines the judgments people make about others based upon how much they eat. Moreover, it is not just amount of food that changes when someone wants to make a good impression. A woman eating with a man eats lower calorie foods than one eating with another woman, and the caloric intake of women in a group eating together is negatively correlated to the number of men in the group [11]. If we use food intake to influence how others view us, clearly we expect others to attend to what we eat as much as we concentrate on their consumption.

The large literature on social modeling of intake also reflects comparing one’s own intake with someone else’s. In the case of modeling of intake, as mentioned earlier, people tend to eat more with someone who eats a lot and less with someone who eats minimally (e.g., [1]). Again, this clearly reflects attentiveness to other people’s intake. Social comparison is rarely mentioned in discussions of factors influencing eating behavior, but we contend that it is actually one of the most critical social influence factors. Social comparison actually seems to underlie both impression management using food and social modeling of intake levels, as they both rest on an assumption that we compare our food intake to each other when we eat together. The individual may even rely on social comparison when eating alone, by comparing one’s own intake to a social norm of what is appropriate to eat (e.g., [12]). So other than straight social facilitation, the main social influences on eating actually stem from social comparisons.

Social comparison can occur both on amounts and types of food eaten as well as on dimensions related to food and eating such as body weight or physique. Eating behavior following both types of social comparisons may be affected. When young women are induced to compare themselves to fashion models (by showing them images from fashion magazines in a way that encourages comparison), they tend to feel worse about themselves and their eating is affected (though differently for chronic dieters/restrained eaters and unrestrained/nondieters) [13, 14].

There is some, though limited, direct evidence of social comparison effects on eating that do not underlie further social effects such as impression management or modeling behavior, and are not related to other dimensions such as body image, but are simply comparisons of our own and other’s eating that have effects on how we eat and how we feel about it. In a study we published several years ago [15], we showed that our female college student participants were attending closely to, and comparing, their own portion of food and that being offered to another participant. We served everyone in our experiment a standard slice of pizza from a popular local pizza chain. But what the different groups of students saw being given to a supposed second participant in a different room varied.

Bearing in mind what we’ve learned as parents through our own children’s reactions to the sizes of portions served to them and their siblings (especially portions of dessert!), we led the study participants to believe that their portion might not be quite the same as what another participant received. Some students saw that the other person was getting a slice 1/3 smaller than they got, and some saw a slice 1/3 larger going to the other participant (and the control group didn’t see another slice, just their own). In fact, of course, there was no other participant, everyone got the same sized slice of pizza, and our interest was only in how our students reacted to what they thought someone else was getting compared to what they themselves were given.

We also assessed which of the students were restrained eaters (that is, chronic dieters who worry about their eating and weight, but tend to break their diets under many provocations), and which were unrestrained eaters (people not concerned about dieting or weight), as these types of individuals generally differ in their reactions to what they (and perhaps others) eat [16, 17]. Restrained eaters are prone to overeating when they believe that they have already “blown” their diets for the day [18, 19], so if they see themselves as having overeaten on the pizza, they could be more likely to eat more food subsequently. Unrestrained eaters, on the other hand, regulate their intake more based on internal sensations and social norms, and thus would be likely to compensate and eat less after what they saw as a large meal [20].

In fact, this is exactly what the data showed, with a significant interaction between restraint and “pizza size” for amount of cookies eaten when we asked them to taste and rate cookies in a subsequent “perceptual taste test.” There was no effect of thinking that they had received a smaller slice on the eating of either group, and all students rated their slice as “just the right size” when they saw it as smaller than the other person’s. But when they thought they had eaten a “larger than normal/desirable” slice, restrained eaters went on to eat more cookies (as they tend to do when they believe that they have broken their diets), while unrestrained eaters “regulated their intake” and reduced their cookie consumption after what they thought was a large slice of pizza. Interestingly, unrestrained eaters were happiest when they got the smaller slice (which was when restrained eaters felt the worst), and restrained eaters were happier when they got the largest slice (probably because now they were being forced by us to eat a lot, and didn’t have to feel guilty about eating a lot, which appears to be what restrained eaters actually would prefer to be able to do!).

What this study showed most clearly was that all participants, restrained and unrestrained alike, were comparing their food portion to what others were getting, and changing their behavior and how they felt about their food and their eating according to this comparison. Remember, they all got exactly the same-sized slice of pizza!

In a recent as yet unpublished replication study from our laboratory (Polivy J, Herman CP, Teeft T. Eating behavior as a function of perceived portion inequity. In Preparation), we gave students the opportunity to, in effect, correct the inequity in the size of the portions we gave them. After they ate their slice of pizza (which half of them believed was smaller than someone else’s slice, versus the control participants who did not have a comparison slice present), we made more pizza available. Male and female restrained eaters, and female unrestrained eaters who were led to believe that they had received the “smaller” slice compensated for having been “short-changed” by eating more of the “extra” pizza available to them than did their respective control groups, who did not feel they had gotten less initially.

Leone, Herman, & Pliner [21] made participants believe that they had just eaten either twice as much or half as much as a (non-existent) partner in the study ate. This manipulation was intended to induce either a positive social comparison (I ate less than she did and thus performed better on the eating task) or negative comparison (I ate more than she did and thus performed worse on the eating task) between the participant and the putative partner she was going to meet later in the experiment. The main dependent measure was how the participants felt about this supposed other student. The outcome was exactly as social comparison theory predicted, in that the participants felt worse when they ate more than the other person than if they “won the competition” by eating less. Moreover, they then distanced themselves from the other person by rating her as a less desirable work partner, less likeable, and less similar to themselves.

We wondered how far these comparisons extend. Is it only size of portion or amount eaten that others attend to, or are other aspects of the meal being compared? Looking further at the literature, a study by Just and Wansink [22] varied the price of a food and examined the effect of different pricing on amount eaten. Giving people a “deal” by giving them a half-price coupon for however much they ate actually led them to eat less pizza (2.95 slices) than those paying full price (4.09 slices), but they liked the cheaper pizza more. So changing the price for some changed their reaction to the food, making them like their bargain more, while eating less of it.

In a conceptual replication of our own pizza slice studies (Polivy J, Herman, CP, Garmenova Y. The effects of hedonic contrast and restraint status on food ratings and consumption. In Preparation), we gave all participants a vegetarian submarine sandwich, but told some that another participant was getting a different meal. We found that participants given the submarine sandwich ate very differently depending on what they believed another participant was getting to eat. Restrained eaters who believed that the other person was getting a more desirable meal (pizza) and they were getting a “worse” meal ate less of their sandwich than the other participants did. Both restrained and unrestrained eaters, however, ate more of the sandwich than the control group did when they thought the other person was given a less preferred meal (plain cheese sandwich), and thus they were getting the “better” meal. In addition, the restrained participants reported liking the submarine sandwich less when they thought another participant was eating pizza (and thus their sandwich was worse) than if they thought that they were both eating the same sandwich; however, it was only the unrestrained eaters who increased their ratings of the “better” meal when they thought the other person was getting a plain cheese sandwich.

Social comparisons during eating occasions thus have important effects on the eaters. Comparing our food to what others are getting to eat affects both how much we like our own meal and how much of it we eat. When we make downward comparisons of our own food versus what others are eating, (i.e., the other person’s food is worse than ours is) we seem to value our meal more highly, which promotes and increases both our ratings and our consumption of it, whereas upward comparisons have the reverse effect. Feeling worse about our meal makes it less desirable and thus we eat less of it. Moreover, if we are able to compare our food to that of others, and emulating them is both desirable and “attainable” (i.e., we want to be able to eat more and we actually can increase our portion size to match that of another eater), we will change our intake accordingly, especially if we are dieters who are highly concerned with how much we and those around us are eating.

Why are we so concerned with what others eat? Apparently the same processes that push us to make upward and downward comparisons of ourselves with others on so many other attributes apply equally well to eating. Our basic drive to evaluate ourselves by comparing ourselves to others, especially similar others like our peers (according to Festinger) extends to what and how much food we eat. Our food comparisons also affect how we feel about co-eaters who are getting more or better food, or reduced food amounts and quality, as Leone et al. showed. This makes sense on many levels (e.g., [17]). If we are eating less food than others, this can make us physically weaker and less fit to survive. If we are getting lower quality/less preferable food, this may also reflect or affect our social status. The king and his court eat more desirable foods than do the peasants. We don’t want to think of ourselves as having lower social status than our peers, so we need to compare and make sure we are eating as well as they are. And if we are happier with our meals and enjoy them more, then our mood is elevated. It is sad but true that people like to feel they are getting a better deal than others or doing better than their peers, and eating is no exception to this.

Thus this social comparison of others’ eating to our own is the basis for many of the phenomena that we think of as social influences on eating [23]. The reason we model another’s intake is because we compare ourselves to the other, and want to maintain our status in the comparison. If our goal is to eat as much as we can without eating to excess (as Herman, Roth, & Polivy, [24], have argued), then we can “win” in the social comparison game by modeling our intake on another eater and eating just a little less than s/he does. Indeed, the modeling literature seems to show just this—we eat more with an augmenting model than with no model or one who eats minimally, but generally intake is lower than the augmenting model ingests. If, on the other hand, the model is eating a minimal amount, people reduce their intake, but may eat a bit more than the model, perhaps to show that while they are appropriately limiting their intake, they “win” by getting a little more than the low-intake model. In either case, our modeling of the intake of other eaters reflects our social comparison with their behavior, and presumably serves a social comparison goal.

Similarly using food for impression management is also based on social comparison. We assume others are watching what we eat, so that we can impress the other observing our eating with our femininity by eating minimally or masculinity by eating more heartily (and, of course, avoiding quiche and other “lady foods”). Thus, social comparison is the requisite basis for at least these two forms of social influence on eating.

## Conclusion

To summarize our thesis, we know from the vast social comparison literature that such comparisons are important for our identity and our evaluations of ourselves. It appears from the present research that we are also very careful to compare our own plates to what others are eating, suggesting that this also provides valuable information. By watching others eat, we learn what and how much to eat, how much we like what we are eating or would prefer something else, how we feel about our own consumption compared to others’ intake, and how we feel about other people who eat similarly or differently from us. Understanding these effects could be helpful in designing treatments for overeating/obesity. For example, having obese people eat with others can be helpful if the others are eating smaller portions. On the other hand, however, those inclined to overeat may be encouraged to do so when eating with others who consume large amounts. Treatment might be aimed at making potential overeaters aware of these influences so that they can plan their own eating without being swayed by others who are eating larger amounts. Eating disorder patients, who are exquisitely sensitive to what those around them are eating, seem to be motivated to eat less than anyone else, and thus it might not be helpful to have them eating with other patients with whom they can compare intake.

As comparing ourselves to others can change not only how we feel but how much we eat, clearly social comparison with respect to eating is worth further study.
